# Azithromycin in Chronic Fatigue Syndrome (CFS), an analysis of clinical data

**DOI:** 10.1186/1479-5876-4-34

**Published:** 2006-08-15

**Authors:** Ruud CW Vermeulen, Hans R Scholte

**Affiliations:** 1 CFS and Pain Research Center Amsterdam, Waalstraat 25-31, 1078 BR Amsterdam, The Netherlands; 2 Department of Biochemistry, Erasmus MC-University Medical Center Rotterdam, Rotterdam, The Netherlands

## Abstract

**Background:**

CFS is a clinical state with defined symptoms, but undefined cause. The patients may show a chronic state of immune activation and treatment with an antibiotic in this subgroup has been suggested.

**Methods:**

In a retrospective study, the response of CFS patients to azithromycin, an antibiotic and immunomodulating drug, has been scored from the patients records and compared with clinical and laboratory data. Azithromycin was not the first choice therapy, but offered when the effect of counseling and L-carnitine was considered insufficient by the patient and the clinician.

**Results:**

Of the 99 patients investigated, 58 reported a decrease in the symptoms by the use of azithromycin. These responding patients had lower levels of plasma acetylcarnitine.

**Conclusion:**

The efficacy of azithromycin in the responsive patients could be explained by the modulating effect on a chronic primed state of the immune cells of the brain, or the activated peripheral immune system. Their lower acetylcarnitine levels may reflect a decreased antioxidant defense and/or an increased consumption of acetylcarnitine caused by oxidative stress.

## Background

In 1994 Fukuda et al. [1] defined CFS as a chronic persistent fatigue that is present for over 6 months, is not caused by activity nor alleviated by rest and accompanied with at least 4 other symptoms: cognitive impairment, pain in joints, muscles or head, unrefreshing sleep, soar throat, tender lymph nodes and postexertional malaise with slow recovery. Nevertheless, CFS remained subject to debate and even the mere existence of the syndrome was still questioned by some. The presentation of the results of quantitative morphology of the brain in CFS patients by Okada et al [2], later confirmed by De Lange et al [3] may change this opinion. The loss of grey matter in the brain, especially in Brodmann's area 9, was related to physical impairment, but not to the duration of the symptoms. Although other explanations were considered as well, this may indicate the occurrence of a major trauma to the brain at the start of the disease. This could also explain the low recovery rate in adults, because repair in adult brain is limited [4].

The severity of the sickness, both the symptoms and the lowered adaptation to physical stress, may fluctuate whenever infections and stress come and go. The Th1 to Th2 cytokine shift in CFS patients will make them more vulnerable to infections [5] and it has been suggested that they have more often chronic infections [6-8]. The predisposition for acquiring CFS may be genetically determined [9,10], the occurrence is influenced by the severity of the immune response [11]. The result is a chronic hyper-oxidative state of sickness [12,13], in the brain itself [14] that cannot be stopped [15]. Preliminary evidence of a relation between post-infectious fatigue and mitochondrial dysfunction indicates a complex response involving acetylcarnitine [16].

The clinical presentation of the symptoms and chances for recovery will depend on the balance between the irreversible loss of grey cells and activation of the immune system. According to De Meirleir et al, the presence of RNase-L and elastase may offer an indication for this balance [17-19]. Several treatment protocols to counteract the immune activation in CFS were presented [18,20,21], but the results were never validated in a double blind study. Such proof is required as a rationale for treatment and provides a basis to understand the pathophysiology of the disease. Comparing the outcome of clinical treatment protocols might add to our knowledge of CFS and its treatment until double blind studies are available [22].

Azithromycin is an antibiotic with immunomodulatory effects [23-25]. This antibiotic has been successfully used during periods of six months of more in other chronic diseases [26-28]. The side-effects are known for long term use and mainly limited to gastro-intestinal cramps. The chances for resistance limit its use to individual patients under close supervision [28]. The drug is relatively inexpensive and extensive laboratory tests for side effects are not necessary. The result of a study in 10 CFS patients during 1 to 2 months was positive [26]. We studied the medical records of CFS patients for clinical and laboratory data related to the outcome of the treatment with azithromycin.

## Methods

In the CFS and Pain Research Center Amsterdam, patients are diagnosed with CFS according to the Fukuda criteria [1]. Treatment protocols are discussed with the patient. One of the treatment protocols that were offered in our Center from April 2000 till August 2005 was azithromycin 500 mg on 3 consecutive days of the week during 6 weeks [29]. Azithromycin was not a first choice therapy, but offered when the effect of counseling [30] and of L-carnitine during more than 3 months [31] was considered insufficient by the patient and the clinician. The period between the L-carnitine medication and the start of azithromycin was always longer than 2 weeks. The selection was not based on symptoms or laboratory indications of immune activity. This treatment was studied in a retrograde evaluation of the clinical records from April 2000 to August 2005. The change in the clinical condition as experienced by the patient (patient rated clinical global impression of change was scored as negative, neutral or positive after a standard interview [32]). The change had to be out of range of the previous fluctuations of the symptoms for scoring as improvement or deterioration. All patients signed an informed consent permitting the use of the data from the clinical records for evaluation and publication.

At the first visit all patients were evaluated according to the protocol of the 1994 consensus meeting [1]. The severity of CFS was determined with the Stanford Health Assessment Questionnaire (SHAQ) [33], the Multidimensional Fatigue Inventory (MFI-20) [34] and the VAS-scale of the McGill Pain Questionnaire [35]. The presence and severity of somatisation, distress, depression and anxiety was scored with the 4 Dimensional Symptom Questionnaire (4DSQ) [36]. Questionnaires for disease specific symptoms were not available at the treatment period [37].

Free carnitine and carnitine esters in plasma were analyzed using tandem mass spectrometry as described by Vreken et al. [38].

Statistical analyses were conducted using the Statistical Package for the Social Sciences (SPSS 14.0 for Windows, Chicago, Ill).

## Results

Of the 99 patients who were treated with azithromycin, 58 (59%) reported an improvement of symptoms. No patients were worse at the end of the treatment. Side effects were minor bowel problems that resolved by dividing the daily dosage of azithromycin.

The majority of patients was female, 88% and 83% in the non-responder and responder group, respectively. The patients who reacted favorably, the responders, were younger, 34.7 ± 11.5 years (mean ± SD) versus 40.4 ± 12.6 (Student's t-test P = 0.023, 95% CI 0.8 and 10.5), but did not differ from the non-responders in fatigue score (MFI-20), somatisation, distress, depression and anxiety (4DSQ), pain score (VAS scale) or physical capacity (SHAQ) (Table 1). Neither the history, nor the physical examination was indicative for the response and the standard laboratory tests did not differ between the groups.

**Table 1:** Mean (Standard deviation) of the non-responders and responders to azithromycin. MFI-20: Multidimensional Fatigue Inventory. 4DSQ: 4 Dimensional Symptom Questionnaire. Pain VAS score: Visual analogue scale of the McGill Pain questionnaire. Stanford HAQ: Stanford Health Assessment Questionnaire. P: probability favoring the absence of a difference of the means of non-responders versus responders (Student-t test).

	Non responders	Responders	P
	Mean (SD)	Mean (SD)	

Age (y)	40.3 (12.7)	34.7 (11.5)	0.023
MFI-20: General fatigue	19.1 (1.4)	18.7 (1.8)	0.267
MFI-20: Physical fatigue	18.0 (2.3)	17.7 (2.7)	0.532
MFI-20: Mental fatigue	16.4 (3.4)	15.2 (4.2)	0.112
MFI-20: Activity	15.5 (3.3)	15.1 (3.8)	0.595
MFI-20: Motivation	11.0 (4.3)	10.1 (3.6)	0.283
4DSQ: Somatisation	0.91 (0.42)	1.11 (1.03)	0.258
4DSQ: Distress	0.89 (0.54)	0.91 (0.51)	0.859
4DSQ: Depression	0.30 (0.47)	0.35 (0.49)	0.585
4DSQ: Anxiety	0.28 (0.35)	0.33 (0.38)	0.481
Pain VAS score	27.8 (23.3)	26.8 (24.2)	0.888
Stanford HAQ	1.15 (0.19)	1.14 (0.28)	0.957

Plasma acetylcarnitine was lower in responders (4.78 ± 1.66 μM) compared to non-responders (5.87 ± 2.55) (P = 0.023, 95% CI 2.3 and 0.1). Plasma acetylcarnitine was 4.70 ± 1.70 μM in responding female patients (n = 48) and 4.61 ± 1.61 μM in responding men (n = 10) (P = 0.884, 95% CI -1.2 and 0.6). These levels were in non-responding female patients (n = 36) 6.04 ± 2.69 μM and in non-responding males (n = 5) 4.88 ± 1.17 μM (P = 0.412, 95% CI -1.7 and 1.4). Because of the absence of statistical difference between the responding females and males and also between the non-responding males and females, we took their values together and calculated that improvement was present in 77% of the patients with plasma acetylcarnitine concentration less than 4.1 μM, in 58% with acetylcarnitine between 4.1 and 6.5 μM and in 31% with acetylcarnitine higher than 6.5 μM. Free and the higher acylcarnitines were not different in responding females and males and non-responding females and males, and were also taken together (Table 2).

**Table 2:** Mean (standard deviation) of free-carnitine and acylcarnitines of non-responders and responders to azithromycin and controls.

Plasma (acyl)carnitine	Non Responders	Responders	P
(μmol/L)	Mean (SD)	Mean (SD)	

Free carnitine	36.6 (9.3)	34.7 (8.3)	0.374
C2-carnitine	5.9 (2.6)	4.8 (1.7)	0.023
C3-carnitine	0.34 (0.12)	0.34 (0.15)	0.970
C4-carnitine	0.23 (0.10)	0.21 (0.11)	0.415
C8-carnitine	0.11 (0.04)	0.08 (0.04)	0.078
C14:1-carnitine	0.07 (0.04)	0.04 (0.03)	0.083
C16:1-carnitine	0.05 (0.04)	0.04 (0.02)	0.323
C18:1-carnitine	0.16 (0.10)	0.16 (0.14)	0.907

## Discussion

Fifty-nine percent of the patients reported improved symptoms of CFS after long-term use of azithromycin. This confirms the results reported by De Becker et al. [18]. It is unlikely that this therapy is due to placebo response, because such a response is low in CFS (19.6%) [39]. Medical history, physical examination, standard laboratory tests (data not shown) and questionnaires for the severity of symptoms (Table 1.) did not differentiate responders from non-responders. The reported change of symptoms differed greatly between patients and validated test for the severity of CFS-related symptoms was not available at that time. Therefore we decide to present an overall impression, based on a standard interview.

The more subtle sign of metabolic (oxidative) stress was reflected by the differences in plasma acetylcarnitine levels[40].

Significant differences in plasma acetylcarnitine [41,42] were reported in CFS, while others found no difference [43,44]. There are several explanations for this discrepancy. One explanation could be that the selection of CFS patients differed. The patients of Kuratsune et al. [41,42] were selected according to the CDC criteria, but Jones et al [43] used the Oxford and CDC criteria.

The responders did not fully recover from CFS by azithromycin. They improved only to an estimated maximum of 80% of the pre-morbid capacity. There are two explanations for the improvement by azithromycin: A reduction of bacterial load [26] or the immune modulating effect of macrolides [23,45]. If CFS is caused by a primed state of glia-cells in the brain [46] in a subgroup of patients, these cells would produce interleukin-1β at an increased rate as response to minor stimulation by stress or infection. The result is a chronic and fluctuating state of sickness that is not fully explained by the presence of an active and detectable disease state. Then it is likely that the immunomodulating drug azithromycin could improve the severity of CFS symptoms.

## Conclusion

Lower plasma acetylcarnitine was observed in CFS patients who responded to azithromycin. The lower plasma acetylcarnitine level may indicate a decreased ability to counteract the action of the increased oxidative stress and/or an increased consumption of acetylcarnitine by the increased oxidative stress.

The response rate of 59% in this retrospective open study is not necessarily indicative for the effect rate in prospective blinded studies.

## Competing interests

The author(s) declare that they have no competing interests.

## Authors' contributions

The investigation was planned by and carried out under the clinical supervision of RCWV. HRS helped by interpretation and selection of the data, and correction of the manuscript.
